# Clinical significance of subclinical carotid atherosclerosis and its relationship with echocardiographic parameters in non-diabetic chronic kidney disease patients

**DOI:** 10.1186/1471-2261-13-96

**Published:** 2013-11-06

**Authors:** Jwa-Kyung Kim, Young Rim Song, Min Gang Kim, Hyung Jik Kim, Sung Gyun Kim

**Affiliations:** 1 Department of Internal Medicine & Kidney Research Institute, Hallym University College of Medicine, 896, Pyeongchon-dong, Dongan-gu, Anyang-si 431-070, Korea

**Keywords:** Carotid atherosclerosis, Cardiovascular events, Non-diabetic chronic kidney disease, Increased left ventricular filling pressure

## Abstract

**Background:**

Non-diabetic chronic kidney disease (CKD) patients are a heterogeneous group with a variety of prognosis. We investigated the role of subclinical carotid atherosclerosis for the prediction of adverse cardiovascular (CV) outcomes in these patients, and tried to identify clinical and echocardiographic parameters associated with subclinical carotid atherosclerosis.

**Methods:**

As a prospective design, 182 asymptomatic non-diabetic CKD patients underwent carotid ultrasonography and Doppler echocardiography. Carotid atherosclerosis was defined as a carotid intima-media thickness ≥1.0 mm and/or the presence of plaque.

**Results:**

During the mean follow-up period of 28.8 ± 16.1 months, 23 adverse CV events occurred. Patients with carotid atherosclerosis (99, 54.4%) showed significantly higher rates of annual CV events than those without (8.6 vs. 1.5%, p <0.001). Particularly, the presence of carotid plaque was a powerful predictor of adverse CV outcomes (OR 7.80, 95% CI 1.45-45.97). Clinical parameters associated with the presence of subclinical carotid atherosclerosis were old age, previous history of hypertension, increased pulse pressure, and higher high-sensitivity C-reactive protein (hs-CRP) level. By echocardiography, early diastolic mitral annular velocity (E’) and the ratio of early peak transmitral inflow velocity (E) to E’ (E/E’) were closely related with the presence of carotid atherosclerosis. A multivariate analysis showed that age, hs-CRP, and E/E’ were significant determinants of carotid atherosclerosis.

**Conclusions:**

Carotid plaque, even subclinical, was closely associated with a poor prognosis in non-diabetic CKD patients. Increased age, hs-CRP level, and E/E’ ratio may be useful markers suggesting the presence of carotid atherosclerosis in these patients.

## Background

Patients with chronic kidney disease (CKD) are often regarded as the highest risk population for cardiovascular (CV) disease. However, CKD patients are a heterogeneous group, and there are subgroups at lower risk for CV complications as well as subgroups with increased need of intensive medical care and close follow-up. Since diabetes has long been identified as a CV disease risk equivalent, patients with diabetic CKD usually have increased CV risk and poor prognosis, as compared to those with non-diabetic CKD. However, the long-term prognoses of non-diabetic CKD patients are especially diverse; therefore, non-invasive and effective risk assessments are particularly needed for this group. Atherosclerosis is the main pathophysiological link between CKD and CV disease, and the presence of carotid atherosclerosis, even subclinical, could be a useful maker predicting adverse CV outcomes [1-4]. To date, current guidelines recommend the use of carotid atherosclerosis in asymptomatic patients at intermediate risk. Therefore, screening for carotid atherosclerosis may be helpful for the identification of high-risk group in non-diabetic CKD patients, although routine screening for carotid atherosclerosis in all CKD patients (including diabetes) cannot be justified.

According to the previous multi-ethnic observational study, subclinical carotid atherosclerosis was closely associated with significant alterations in myocardial strain parameters, reflecting incipient myocardial systolic and diastolic dysfunction [5]. However, no data regarding the relationship between echocardiographic parameters and carotid atherosclerosis have been reported in these patients.

In this study, we examined the impact of subclinical carotid atherosclerosis, as measured by carotid intima-media thickness (cIMT) and carotid plaque, on long-term CV outcomes in asymptomatic non-diabetic CKD patients, and tried to identify clinical and echocardiographic parameters suggesting the presence of subclinical carotid atherosclerosis in these patients.

## Methods

### Population

Since January 2008, our hospital began a CV surveillance program for asymptomatic stable CKD patients. CKD is defined as low estimated glomerular filtration rate (eGFR) < 60 mL/min/1.73 m2 with abnormalities of kidney structure or function, present for > 3 months with implications for health. To avoid the enrollment of ineligible patients, those taking any medications that may affect renal function, such as non-steroidal anti-inflammatory drugs, antibiotics, and herbal medications at the time of evaluation were not included. Demographic, laboratory, and clinical data including classic CV risk factors were obtained. Additionally, resting ECG, Doppler echocardiography (Vivid 7; GE Medical, Horton, Norway), and carotid B-mode ultrasonography (US) (Vivid 7; GE Medical, Horton, Norway) were performed with patient consent. Two hundred and thirty-four patients were registered in our cohort between January 2008 and December 2012. Fifty-two patients were excluded for the following reasons: incomplete data from echocardiography or carotid US (*n* = 11), previously diagnosed atherosclerotic diseases such as cerebrovascular accidents (*n* = 7), coronary artery disease (*n* = 13), peripheral arterial disease (*n* = 2), presence of edema at the time of echocardiography (*n* = 12), or previously diagnosed cardiomyopathy (*n* = 7). Consequently, data from 182 patients were analyzed, and informed consent was obtained from each patient. This study was approved by the Hallym University Sacred heart hospital Institutional Review Board/Ethics committee and conducted according to the Declaration of Helsinki.

### Diagnostic work-up

All echocardiographic images were obtained with standard techniques using the M-mode, 2D, and Doppler measurements in accordance with the American Society of Echocardiography guidelines. LV systolic function was assessed by LV ejection fraction (LVEF) using the modified biplane Simpson’s method with apical two- and four-chamber views. The LV mass was calculated according to the recommended ASE formula and indexed for body surface area (BSA) (LVMI, g/m2). LV diastolic filling pattern was assessed by the transmitral inflow velocity curve, including peak E velocity (peak transmitral velocity during early diastole), deceleration time (DT), peak A velocity (peak transmitral atrial filling velocity during late diastole), and E/A ratio. For tissue Doppler images, 2.0-mm sample volume was sequentially placed at septal and lateral annular sites and the average of the two values was used to evaluate the early (E’) and late (A’) diastolic mitral annular velocities. Filters and gains were adjusted to minimize background noise and maximize tissue signal, and the E/E’ ratio was also obtained. The left atrial (LA) volume was also measured from the apical two- and four-chamber views at end-systole by tracing the LA borders using planimetry. The LA volume index (LAVI) was calculated by dividing the LA volume by BSA.

Ultrasonographical B-mode imaging of bilateral carotid arteries was also performed with high resolution real-time US with a 12-MHz linear-assay transducer. Bilateral carotid arteries, carotid bulbs, and internal carotid arteries were examined by two different longitudinal projections, and 2D images were acquired on the R wave of the electrocardiography (ECG), frozen in end-diastole and analyzed off-line. The cIMT was defined as the distance between the leading edges of the lumen interface and the media-adventitia interface at the far wall in plaque-free arterial segments. Carotid plaque was defined as a focal structure encroaching into the arterial lumen of at least 0.5 mm or 50% of the surrounding IMT value, or demonstrates a thickness >1.5 mm as measured from the media-adventitia interface to the intima-lumen interface [6]. Carotid atherosclerosis was defined as carotid intima-media thickness (cIMT) ≥1.0 mm and/or the presence of plaque.

### Follow-up and endpoints

Patients were followed by periodic examination in an outpatient setting. For patients not followed at our center, information was obtained by a telephone interview. The end of follow-up was determined by the occurrence of adverse CV events or by the date of last patient contact in the hospital for those without events. CV events were defined as fatal or non-fatal acute coronary syndrome (ACS) (e.g., acute myocardial infarction and unstable angina), or cerebrovascular events. ACS was defined using the standard criteria of history, ECG, and cardiac enzyme levels. In cases of multiple cardiac events, only the first event was used as the end point of follow-up.

### Statistical analysis

Statistical analyses were performed using SPSS version 18.0 (SPSS Inc., Chicago, IL, USA). All variables were expressed as means ± standard deviations (SDs) or medians with ranges unless otherwise indicated. The Kolmogorov-Smirnov test was used to analyze the normality of distributions, and natural log values were used for skewed data. Correlations between clinical or echocardiographic factors and carotid atherosclerosis were evaluated using Spearman’s rank correlation. By multiple regression analysis, the effects of clinical and echocardiographic parameters on the presence of carotid atherosclerosis were evaluated and an area under the receiver operating characteristic (ROC) curve was used to compare various echocardiographic parameters for predicting carotid atherosclerosis. Survival curves were derived by the Kaplan-Meier method; the differences between survival curves were compared using the log-rank test. The multivariate Cox proportional hazard model was used to evaluate independent predictors of adverse CV outcomes. A p-value <0.05 was considered statistically significant.

## Results

A total of 182 patients (mean age, 67.8 ± 14.7 years; males, 96 [52.7%]) were analyzed. Subclinical carotid atherosclerosis was found in 99 patients (54.4%); the demographic and biochemical characteristics of the patients with and without carotid atherosclerosis are compared in Table 1. The mean cIMT value of the patients with carotid atherosclerosis was 0.95 ± 0.18 mm, and 90.9% of them had carotid plaque. The group with carotid atherosclerosis was significantly older and had a higher prevalence of hypertension and increased pulse pressure. In addition, serum phosphorus level was significantly lower and low-density lipoprotein (LDL) cholesterol, triglyceride, and high-sensitivity C-reactive protein (hs-CRP) levels were higher in these patients. However, there were no differences in the amount of proteinuria or the prevalence of statin use between the two groups.

**Table 1:** Clinical characteristics of the study population

**Clinical characteristics**	**Total (n = 182)**	**Carotid atherosclerosis**		
		**+ (n = 99, 54.4%)**	**- (n = 83, 45.6%)**	***p***
Age (years)	67.8 ± 14.7	74.5 ± 9.8	59.9 ± 15.6	<0.001
Male, *n* (%)	96 (52.7)	54 (54.5)	42 (50.6)	0.351
Smoker, *n* (%)	70 (38.4)	41 (41.4)	29 (34.9)	0.756
Body mass index (kg/m^2^)	24.1 ± 3.4	23.6 ± 3.1	24.7 ± 3.8	0.065
Hypertension, n (%)	129 (70.8)	81 (81.8)	48 (57.8)	0.001
Systolic blood pressure (mmHg)	134.3 ± 21.0	136.0 ± 23.4	131.5 ± 17.4	0.093
Diastolic blood pressure (mmHg)	75.5 ± 12.0	73.7 ± 12.6	77.6 ± 10.8	0.026
Pulse pressure (mmHg)	58.8 ± 17.1	62.9 ± 18.9	53.9 ± 13.1	<0.001
Baseline laboratory findings				
Hemoglobin (g/dL)	11.3 ± 2.1	11.5 ± 2.0	11.3 ± 2.2	0.553
Albumin (g/dL)	3.9 ± 0.6	3.8 ± 0.6	4.0 ± 0.6	0.144
Blood urea nitrogen (mg/dL)*	28.7 (10.8-118.3)	30.9 (10.8-118.3)	27.1 (12.2-99.0)	0.065
Creatinine (mg/dL)	2.6 ± 2.5	2.7 ± 2.0	2.4 ± 1.7	0.229
eGFR (mL/min/1.73 m^2^)	28.1 ± 16.1	23.6 ± 15.5	30.1 ± 18.5	0.185
Calcium (Ca)	8.8 ± 0.7	8.8 ± 0.6	8.6 ± 0.8	0.128
Phosphorus (P)	3.7 ± 1.2	4.0 ± 1.4	3.5 ± 0.9	0.030
Ca * P product	34.6 ± 9.4	35.4 ± 18.3	34.2 ± 10.8	0.292
Total cholesterol (mg/dL)	163.1 ± 44.8	169.8 ± 41.3	157.6 ± 47.1	0.092
HDL-cholesterol (mg/dL)	47.0 ± 15.2	47.9 ± 16.5	45.9 ± 12.9	0.456
LDL-cholesterol (mg/dL)	99.8 ± 35.2	108.9 ± 35.8	93.2 ± 33.3	0.011
Triglyceride (mg/dL)	112.9 ± 51.7	128.2 ± 51.3	101.6 ± 49.4	0.003
Urine PCR†	-1.17 ± 1.42	-1.34 ± 1.27	-0.98 ± 1.57	0.103
hs-CRP (mg/L) †	-0.20 ± 1.12	-0.01 ± 1.19	-0.43 ± 0.98	<0.001
Statin use, n (%)	105 (57.6)	61 (61.6)	44 (53.0)	0.041
cIMT (mm)	0.87 ± 0.18	0.95 ± 0.18	0.75 ± 0.09	<0.001
Plaque, n (%)	90 (49.5)	90 (90.9)	-	-

*Abbreviations*: HDL, High-density lipoprotein; LDL, Low-density lipoprotein; urine PCR, Urine protein-to-creatinine ratio. *, median with ranges. †, log-transformed.

### Carotid atherosclerosis and its relationship with echocardiographic parameters

Differences in various echocardiographic parameters according to the presence of carotid atherosclerosis are shown in Table 2. The prevalence of LV hypertrophy was higher in patients with carotid atherosclerosis (43.4 *vs*. 31.3%, *p* = 0.043); however, the LV systolic and diastolic volume indices, LVEF, LVMI and LAVI were similar between the two groups. Interestingly, pronounced differences were found in markers of elevated LV filling pressure between the two groups; the E/A ratio (0.72 ± 0.22 *vs*. 0.83 ± 0.32, *p* = 0.032) was significantly lower while the DT (239.2 ± 46.4 *vs*. 219.3 ± 46.4, *p* = 0.019) was longer in patients with carotid atherosclerosis than in patients without. Furthermore, tissue Doppler echocardiography showed similar findings: E’ (5.6 ± 2.2 *vs*. 6.6 ± 1.9, *p =* 0.003) was lower, but the E/E’ ratio (12.5 ± 4.4 *vs*. 10.2 ± 3.1, *p* <0.001) was higher in patients with carotid atherosclerosis (Table 2).

**Table 2:** Differences in echocardiographic parameters according to the presence of carotid atherosclerosis

**Clinical characteristics**	**Total (n = 182)**	**Carotid atherosclerosis**		
		**+ (n = 99, 54.4%)**	**- (n = 83, 45.6%)**	***p***
LV end-diastolic diameter (mm)	47.3 ± 5.7	46.9 ± 6.7	47.7 ± 4.2	0.392
LV end-systolic diameter (mm)	31.7 ± 5.6	31.5 ± 6.1	32.1 ± 5.2	0.555
LV end-diastolic volume index (mL/m^2^)	52.1 ± 9.1	48.9 ± 12.8	55.6 ± 13.3	0.109
LV end- systolic volume index (mL/m^2^)	21.3 ± 9.2	20.6 ± 9.2	22.2 ± 9.1	0.373
LVMI (g/m^2^)	99.3 ± 29.5	94.3 ± 27.3	101.0 ± 31.2	0.170
LVEF (%)	59.1 ± 9.7	58. 0 ± 11.0	60.4 ± 7.7	0.161
LAVI (mL/m^2^)	42.2 ± 17.3	42.9 ± 17.7	39.5 ± 16.9	0.191
LVH, n (%)	69 (37.9)	43 (43.4)	26 (31.3)	0.043
Mitral inflow velocities (mean ± SD)				
E (cm/s)	64.7 ± 19.0	64.1 ± 19.2	70.3 ± 18.9	0.101
A (cm/s)	84.3 ± 20.4	85.5 ± 18.6	82.9 ± 22.2	0.439
E/A ratio	0.77 ± 0.28	0.72 ± 0.22	0.83 ± 0.32	0.032
DT (ms)	231.0 ± 48.0	239.2 ± 46.4	219.3 ± 46.4	0.019
Tissue Doppler parameters				
E’ (cm/s)	6.1 ± 2.1	5.6 ± 2.2	6.6 ± 1.9	0.003
A’ (cm/s)	8.9 ± 1.9	8.7 ± 2.2	9.2 ± 1.6	0.090
E/E’ ratio	11.5 ± 4.1	12.5 ± 4.4	10.2 ± 3.1	<0.001

*Abbreviations*: LVMI, LV mass index; LVEF, LV ejection fraction; LAVI, Left atrial volume index; E, Early peak transmitral inflow velocity; A, late diastolic transmitral inflow velocity; DT, Deceleration time; E’, Early diastolic mitral annular velocity; A’, Late diastolic mitral annular velocity.

**Figure 1:**
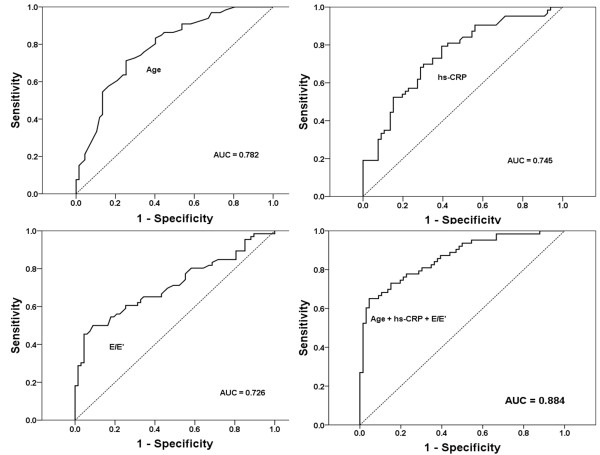
**ROC curves for age, hs-CRP, and the E/E’ ratio for the prediction of carotid atherosclerosis.** When age, hs-CRP and the E/E’ ratio were combined, the AUC significantly increased to 0.884.

According to the results of correlation analysis, carotid atherosclerosis had a strong positive association with age (*r* = 0.486, *p* <0.001), history of hypertension (*r* = 0.228, *p* = 0.002), pulse pressure (*r* = 0.238, *p =* 0.001), and hs-CRP level (*r* = 0.436, *p* <0.001). For the echocardiographic parameters, carotid atherosclerosis was closely associated with the E/A ratio (*r* = - 0.168, *p* = 0.035), E’ (*r* = - 0.245, *p =* 0.001), A’ (*r* = - 0.209, *p =* 0.009) and E/E’ ratio (*r* = 0.339, *p* < 0.001). Using these results, a stepwise multiple regression analysis showed that age (β = 0.02, *p* <0.001), hs-CRP level (β = 0.08, *p* = 0.013) and E/E’ ratio (β = 0.02, *p* = 0.034) were significant determinants of carotid atherosclerosis in non-diabetic CKD patients, even after adjustments for other well-known CV risk factors (Table 3). The area under the ROC curve of age, hs-CRP and E/E’ ratio to predict carotid atherosclerosis were 0.782 (0.690 - 0.851), 0.745 (0.661 - 0.830), and 0.726 (0.623-0.801), respectively. When age, hs-CRP and E/E’ were combined, the AUC significantly increased to 0.884 (Figure 1).

**Table 3:** Clinical and echocardiographic parameters associated with carotid atherosclerosis in non-diabetic CKD patients

**Parameters**	**Univariate analysis**		**Multivariate analysis**	
	**Unstandardized coefficient**	**P**	**Unstandardized coefficient**	**P**
Age	0.02 (0.01, 0.02)	<0.001	0.02 (0.01, 0.02)	<0.001
Hypertension	0.29 (0.11, 0.48)	0.002	0.12 (-0.06, 0.30)	0.199
Systolic BP (mmHg)	0.01 (-0.01, 0.01)	0.103	-	-
Diastolic BP (mmHg)	-0.01 (-0.02, -0.01)	0.026	-0.01 (-0.06, 0.31)	0.177
Pulse pressure (mmHg)	0.01 (0.01, 0.02)	<0.001	0.01 (-0.01, 0.01)	0.267
BMI (kg/m^2^)	-0.01 (-0.04, 0.01)	0.176		
Urine PCR (g/g)*	-0.04 (-0.10, -0.01)	0.097	-	-
hs-CRP*	0.09 (0.02, 0.15)	0.010	0.08 (0.02, 0.14)	0.013
Statins	0.11 (-1.10, 1.26)	0.215	-	-
Echocardiographic data				
LVH	0.13 (-0.02, 0.28)	0.095	-	-
LVEF	-0.01 (-0.01, 0.01)	0.161	-	-
E/A ratio	-0.28 (-0.56, 0.01)	0.052	-	-
E’	-0.05 (-0.08, -0.02)	0.003	-	-
E/E’ ratio	0.04 (0.02, 0.06)	<0.001	0.02 (0.01, 0.04)	0.034

*, log-transformed.

### Risk factors for long-term CV outcomes

**Figure 2:**
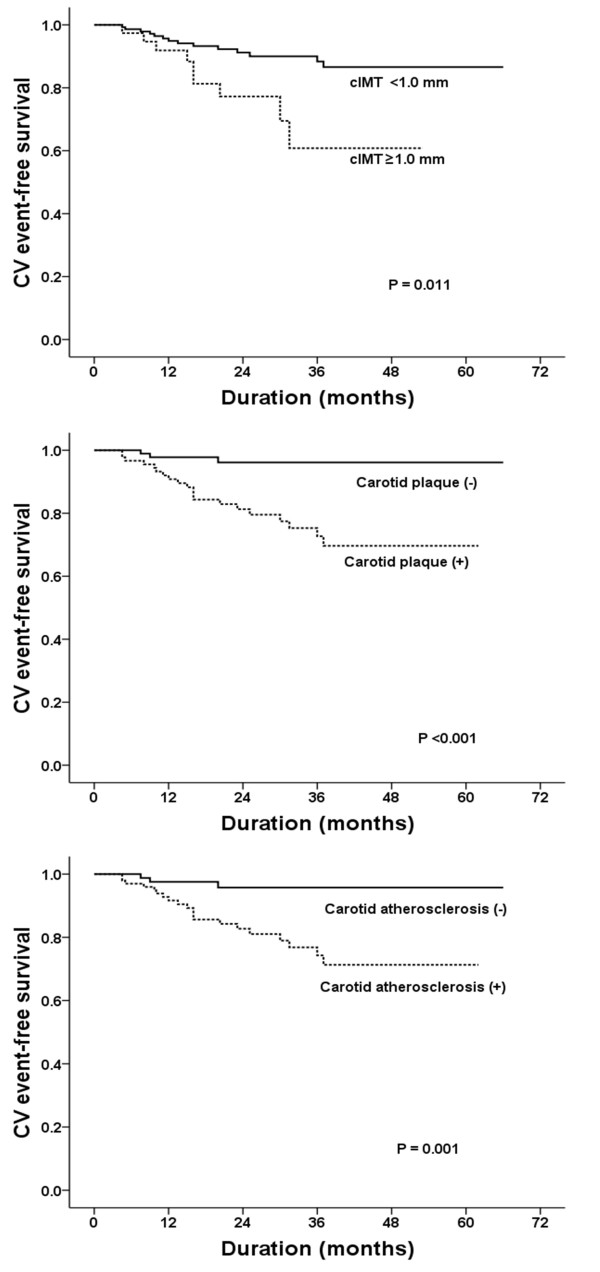
**Cardiovascular event-free survival in analyses by cIMT, carotid plaque and carotid atherosclerosis.** The presence of carotid atherosclerosis was significantly associated with poor prognosis in non-diabetic CKD patients.

During the mean follow-up period of 28.8 ± 16.1 months, 23 adverse CV events occurred: 20 cases with and 3 cases without carotid atherosclerosis. Fatal CV events were occurred in 3 cases at 20.3, 30.3 and 31.5 months of follow-up. The overall annual CV event rate per person-year of follow-up was 5.3%, and the rate was significantly higher in patients with carotid atherosclerosis than in those without (8.6 vs. 1.5%, hazard ratio 5.8, log rank *p* = 0.004) (Figure 2).

To investigate the effect of subclinical carotid atherosclerosis on the occurrence of adverse CV outcomes, we performed a Cox regression analysis. By univariate analysis, smoking, history of hypertension, LVH, increased E/A, E/E’ ratios, carotid plaque, and cIMT values were all significant predictors. After adjustment for these factors, only smoking (OR 3.13, 95% CI 1.10-9.09), an increased E/E’ ratio (OR 1.10, 95% CI 1.01-1.21), and the presence of carotid plaque (OR 7.80, 95% CI 1.45-45.97) were significant independent predictors of CV events (Table 4). Considering that cIMT lost its significance in our multivariate analysis, the presence of carotid plaque is thought to be the main factor causing adverse CV events in patients with carotid atherosclerosis.

**Table 4:** Risk factors for adverse CV outcomes among non-diabetic CKD patients

**Parameters**	**Adverse CV events**		**Univariate analysis**	**Multivariate analysis**	
	**+ (n = 23)**	**- (n = 159)**	**P**	**HR (95% CI)**	**P**
Age (years)†	72.5 ± 11.2	67.2 ± 15.1	0.098	1.05 (0.91-1.01)	0.087
Male, *n* (%)	13 (56.5)	83 (52.2)	0.740	-	-
Smokers, *n* (%)†	13 (56.5)	57 (35.8)	0.012	3.13 (1.10-9.09)	0.036
Hypertension, n (%)†	20 (87.0)	109 (68.5)	0.043	1.61 (0.88-2.51)	0.101
SBP (mmHg)	138.9 ± 31.0	133.6 ± 19.2	0.181	-	-
DBP (mmHg)	78.0 ± 14.0	75.1 ± 11.7	0.150	-	-
Pulse pressure (mmHg)	60.9 ± 24.6	58.5 ± 15.8	0.552	-	-
BMI (kg/m^2^)	24.1 ± 3.2	24.3 ± 3.7	0.746		
Serum creatinine (mg/dL)	2.8 ± 3.1	2.7 ± 2.4	0.910	-	-
LDL-cholesterol (mg/dL)	100.5 ± 34.1	96.2 ± 41.0	0.684	-	-
Urine PCR*	-0.97 ± 1.22	-1.2 ± 1.45	0.426	-	-
hs-CRP (mg/L) *†	0.03 ± 1.27	-0.23 ± 1.10	0.220	1.22 (0.58-1.28)	0.486
Echocardiography					
LVH†	13 (56.5)	56(35.2)	0.029	2.01 (0.81-5.34)	0.128
LVEF	58.3 ± 9.8	59.1 ± 9.7	0.778	-	-
LVMI	112.0 ± 27.9	101.4 ±29.5	0.133	-	-
E/A ratio†	0.64 ± 0.12	0.78 ± 0.30	0.013	0.02 (0.001-0.55)	0.022
E/E’ ratio†	13.7 ± 5.21	11.3 ± 3.7	0.003	1.10 (1.01-1.21)	0.042
Carotid plaque, n (%)†	18 (78.3)	72(45.3)	0.001	7.80 (1.45-45.97)	0.017
Carotid IMT (mm) †	0.95 ± 0.13	0.84 ± 0.15	0.010	10.58 (0.43-26.87)	0.149

†, p <0.05 between patients with and without adverse CV events. *, log-transformed.

## Discussion

In this study, we examined the long-term outcomes and predictors of subclinical carotid atherosclerosis among non-diabetic, pre-dialysis CKD patients. With a prevalence of 54.4%, carotid atherosclerosis was an independent risk factor for adverse CV outcomes. In particular, carotid plaque increased the risk of CV events by 7.8 times. Age, hs-CRP level, and E/E’ ratio could be significant determinants suggesting the presence of subclinical carotid atherosclerosis in these patients.

The number of non-diabetic CKD patients continues to grow owing to the increasing number of elderly people and the recent obesity epidemic. Although CKD itself is considered to be a powerful determinant of CV mortality, non-diabetic CKD patients are a heterogeneous group with a variety of prognoses. The need for effective risk stratification assumes unprecedented significance in these patients, and identification of carotid atherosclerosis, even subclinical, could be a useful tool for prediction of future prognosis.

The prevalence of subclinical carotid atherosclerosis is <5% in healthy young, and 5-10% among the elderly people [7]. In certain populations, however, the prevalence becomes much higher: 15-25% in subjects with hypertension or PAD [8,9], and up to 70% in patients with CAD [10]. Our data showed a fairly high prevalence of subclinical carotid atherosclerosis in non-diabetic CKD patients; about half of the patients (54.4%) had carotid atherosclerosis, and most of them had carotid plaque. Individuals with carotid atherosclerosis had significantly higher rates of adverse CV events compared to patients without. These findings are in keeping with those of previous studies, which demonstrated the harmful effect of carotid atherosclerosis on the risk of CV mortality and morbidity. However, we additionally found that carotid plaque, not cIMT, was a powerful predictor of CV outcomes. The presence of carotid plaque was associated with a 7.8 times higher risk of CV events, whereas cIMT lost its significance in our multivariate analysis. These findings can be explained by the fact that the main causes of non-diabetic CKD in our population were hypertension, which is a main determinant of increasing cIMT. In fact, the value of cIMT as a marker of generalized atherosclerosis has recently been called into question since the primary predictors of increased cIMT are age and hypertension, which do not necessarily reflect the atherosclerotic process [11,12]. Moreover, the 2007 European guidelines on hypertension have emphasized that treatment-induced regression of asymptomatic organ damage could reduce the risk of fatal and nonfatal CV events. This has been shown with the treatment-induced regression of electrocardiographic LVH [13], the echocardiographic LVH [14], LVM and left atrial size [15]. However according to a recent analysis of the ELSA study, the treatment-induced cIMT changes in the carotid arteries failed to document a predictive value for CV events [16]. Therefore, the use and enthusiasm for cIMT as a marker of subclinical organ damage has considerably declined over the last years. Carotid plaque, in contrast, is regarded as a more representative marker of atherosclerotic burden, and it may have greater predictive power for CV outcomes [17].

In our study, carotid plaque, a representative marker of atherosclerosis, was a main predictor of adverse CV events in non-diabetic CKD patients who were not yet on dialysis treatment. This finding would support the results of SHARP (Study of Heart And Renal Protection) study which evaluated the anti-atherogenic effect of statin in large cohort of patients with pre-dialysis CKD and patients undergoing dialysis. The study showed a significant reduction in major CV events [18] with statin treatment, suggesting that atherosclerosis does play an important role for adverse CV events. However, according to the results of another two large clinical trials conducted only in patients receiving hemodialysis, 4D (Deutsche Diabetes Dialyse Studie) [19] and AURORA (A study to evaluate the Use of Rosuvastatin in subjects On Regular hemodialysis: an Assessment of survival and cardiovascular e) [20], statins showed little or no benefit as primary CV diseases prevention. With these, the pathophysiology of CV disease in non-dialysis CKD patients seems to be more strongly associated with atherosclerosis compared to that of patients receiving dialysis treatment. This may be why the carotid plaque predicted adverse CV events in our study.

We then identified clinical and echocardiographic parameters suggesting the presence of subclinical carotid atherosclerosis in our patient group. According to our results, increasing age, a high hs-CRP level, and a high E/E’ ratio were closely associated with the presence of subclinical carotid atherosclerosis. Interestingly, we found that markers of increased LV filling pressure such as the E/A ratio, DT, E’ and E/E’ ratio were closely associated with carotid plaque as well as cIMT. In particular, the effect of an elevated E/E’ ratio on the presence of carotid atherosclerosis was independent of other CV risk factors, including old age, hypertension, high pulse pressure, hs-CRP level, and the use of statins. Indeed, the E/E’ ratio is a representative marker of increased LV filling pressure and is usually associated with LV diastolic dysfunction, ultimately leading to LV systolic dysfunction and overt heart failure [21,22]. In fact, several longitudinal studies have revealed that the LAVI [23] and E/E’ ratio [24,25] can serve as independent predictors of CV and all-cause mortality in dialysis patients. Similarly, in our study, the E/E’ ratio was an independent risk factor for both carotid atherosclerosis and poor CV outcomes. The close association between the E/E’ ratio and carotid atherosclerosis explain why an elevated E/E’ ratio is a strong and independent predictor of adverse CV outcomes in various populations.

Several pathophysiologic mechanisms can be proposed for the link between a high LV filling pressure and carotid atherosclerosis. First, high blood pressure might be one of them. The findings noted on transmitral flow and tissue Doppler findings may be reflective of more significant hypertensive heart disease; thus, the link may be hypertension causing abnormalities in the echocardiographic parameters and contributing to the development of carotid disease. Another possible explanation is vascular stiffness. As our data show, those patients with carotid atherosclerosis had a significantly higher pulse pressure than did their counterparts. In patients with end-stage renal disease, increased vascular stiffness and a resultant higher brachial-ankle pulse wave velocity was closely associated with advanced carotid atherosclerosis [26]. Increased vascular stiffness is an important aggravating factor in LV systolic and diastolic dysfunction leading to a rise in LV filling pressure [27]. Third, chronic low-grade inflammation may be related to the increase in LV filling pressure and carotid atherosclerosis. Inflammatory cytokines such as interleukin-6 (IL-6), tumor necrosis factor-α, and hs-CRP are well-established markers of carotid atherosclerosis [28,29]. Such inflammatory cytokines could induce LV diastolic dysfunction or even diastolic heart failure via effects on both myocyte contractility and the extracellular matrix [30].

The current study has several limitations. First, the causal relationship between carotid atherosclerosis and increased LV filling pressure could not be precisely identified. Because both LV filling pressure and cIMT are known to arise in early-stage kidney disease, accelerated atherosclerosis could affect on the increase in LV filling pressure. A long-term follow-up study is needed to address this issue. Second, as this study was conducted at a single hospital, our results are not validated or replicated for any other data set. In addition, although we included only stable CKD patients, the possibility of acute renal deterioration during study period or the frequency of renal function measures may be one of the limitations of our study. Third, although patients with a clinically apparent volume overload status were excluded, long-standing subclinical volume overload may be present in CKD patients, and volume-overloaded hearts could have been misclassified as having increased LV filling pressure. At last, a relatively small size of population is a limitation of our study.

## Conclusions

Subclinical carotid atherosclerosis is common in non-diabetic CKD patients and closely associated with adverse CV events. In particular, carotid plaque was an independent prognostic factor for long-term CV outcomes. Along with such well-known risk factors as old age and higher hs-CRP level, increased E/E’ ratio was an important predictor suggesting the presence of carotid atherosclerosis in non-diabetic CKD patients.

## Abbreviations

ACS: Acute coronary syndrome; BSA: Body surface area; CKD: Chronic kidney disease; CV: Cardiovascular; cIMT: Carotid intima-media thickness; DT: Deceleration time; eGFR: Estimated glomerular filtration rate; ECG: Electrocardiography; hs-CRP: High-sensitivity C-reactive protein; US: Ultrasonography; LVEF: LV ejection fraction; LVMI: LV mass index; LA: Left atrial; LAVI: LA volume index; E: Peak transmitral velocity during early diastole; A: Peak transmitral atrial filling velocity during late diastole; E’: Early diastolic mitral annular velocity.

## Competing interests

There is no conflict of interest in this study. No funding in this study.

The English in this document has been checked by at least two professional editors, both native speakers of English. For a certificate, please see: http://www.textcheck.com/certificate/EIYCIL.

## Authors’ contributions

J-KK data collection, analysis and writing up, YRS data analysis and statistical advisory, MGK data analysis and statistical advisory, HJK study design determination, SGK research initiative and study design determination. All authors read and approved the final manuscript.

## Pre-publication history

The pre-publication history for this paper can be accessed here:

http://www.biomedcentral.com/1471-2261/13/96/prepub
